# Examining the effect of personality on user acceptance of conditionally automated vehicles

**DOI:** 10.1038/s41598-024-84776-4

**Published:** 2025-01-07

**Authors:** S. Nordhoff, E. Lehtonen

**Affiliations:** 1 Department Transport & Planning, Delft University of Technology, Delft, The Netherlands; 2 Electric Vehicle Research Center, Institute of Transportation Studies, University of California, Davis, USA; 3 VTT Technical Research Centre of Finland Ltd, P.O. Box 1000, VTT, FI-02044 Finland

**Keywords:** User acceptance, Conditionally automated driving, UTAUT2, Personality, Big five, Psychology, Human behaviour

## Abstract

Automated vehicle acceptance (AVA) research has grown substantially in the past few years. There is a paucity of research on the role of the big five personality traits on attitudes towards automated vehicles (AVs) and AVA. This is a critical shortcoming given that personality is considered a critical factor explaining technology adoption. Our major theoretical contribution is the integration of the most popular personality measure – the big five – and one of the most influential technology acceptance models – Unified Theory of Acceptance and Use of Technology (UTAUT2). A questionnaire was administered to 9,339 respondents from nine countries to predict the behavioral intention to use conditionally automated vehicles (CondAVs). The original UTAUT2 was extended by trust and driver engagement and the big five personality traits openness, conscientiousness, extraversion, agreeableness, and neuroticism. Structural equation modeling was applied to examine the direct effects of these constructs on behavioral intention and the indirect effects of the personality traits on the independent constructs of the extended UTAUT2. The results have shown positive effects of social influence, trust, and performance expectancy on the behavioral intention to use CondAVs. Most of the hypotheses pertaining to the role of the personality traits on the UTAUT2 constructs were supported, but the effects were relatively small (< 0.25). Our findings support the usefulness of UTAUT2 in evaluating the success of AVs, providing crucial insights into the factors driving the acceptance of CondAVs. The cross-country analysis provides further insights into the role of an individual’s personality for AVA. Our study yields important implications for practitioners. Given the small effect sizes of personality, designing CondAVs around the personalities of their customers during development and commercialization may be ineffective to promote trust and acceptance.

**Supplementary Information:**

The online version contains supplementary material available at 10.1038/s41598-024-84776-4.

## Introduction

The field of automated vehicle acceptance (AVA) has gained enormous interest in the past few years. Understanding why and how people accept and use automated vehicles (AVs) is extremely important to ensure that the promise of a more sustainable and livable future with automated, electric, shared, and connected vehicles is kept. Therefore, conceptual acceptance models for AVs were developed to explain and predict AVA. Some of these AVA models are rooted in established technology acceptance models, such as the UTAUT2 (Unified Theory of Acceptance and Use of Technology) that was developed to explain technology adoption in the consumer context^1^. UTAUT2 represents one of the most comprehensive technology acceptance models to date, synthesizing eight popular technology acceptance models^2^. Therefore, it “represents a shift from a fragmented view of technology acceptance to a unified view that integrated the major theories and technology acceptance models into a single theory”^3^, p. 495]. Moreover, it has been successfully applied in many previous studies explaining the adoption of AVs, explaining a relatively large amount of variance in the behavioral intention to use AVs, suggesting that it captures one the most relevant variables explaining the adoption of AVs [see ^4^].

UTAUT2 theorizes that technology acceptance, herein defined as the behavioral intention to use, is influenced by performance expectancy (or perceived usefulness) and effort expectancy (or perceived ease of use of the technology), social influence, the availability of facilitating conditions supporting technology use, and hedonic motivation (or the perceived enjoyment)^1^. Age, gender, and experience moderate the relationships between the independent and dependent variables in the model^1,5^. In^6^ the cross-country analysis has revealed that the behavioral intention to use conditionally automated vehicles (CondAVs) is influenced by social influence, performance expectancy, and trust. The influence of facilitating conditions, hedonic motivation, driver engagement, and the moderating effects of age and gender was not significant in most countries. A detailed description of the constructs is provided in this paper.

### Research objectives

UTAUT2 does not consider the impact of personality on the factors in UTAUT2^7^. Personality represents the consistent and stable ways individuals adapts to their environment^8^. Personality is described as a highly stable predisposition towards a behavior or object, which is less prone to changes than an individual’s attitude^9^. An individual’s personality can be measured in different ways. The big five is the most used personality measure^10,11^. It is based on five personality traits. These include openness, conscientiousness, extraversion, agreeableness, and neuroticism.

There is a paucity of research on the role of the big five personality traits on attitudes and AVA^10,11^. This is a major shortcoming given that personality is considered a critical factor explaining technology adoption^10^.

The main objective of the present study is to examine the direct and indirect effects of the big five personality traits openness, conscientiousness, extraversion, agreeableness, and neuroticism on the independent variables (i.e., performance expectancy, facilitating conditions, social influence, hedonic motivation, trust, driver engagement), and the dependent variable (i.e., behavioral intention) in our extended UTAUT2.

To address the research objectives, we applied structural equation modeling (SEM). SEM allows researchers to simultaneously estimate complex relationships among several independent and dependent variables. In contrast to techniques such as multiple regression or analysis of variance, SEM enables a more precise measurement of the study variables as it accounts for measurement error^13^. Its suitability to estimate the relationships between independent and dependent variables is reflected in the bulk of literature applying it to understand the role of different factors predicting technology adoption [see^12^].

### Scientific and practical contributions

Our major theoretical contribution is the integration of one of the most used personality measures – the big five – and one of the most influential technology acceptance models – UTAUT2. In this way, we contribute to knowledge generation on the role of an individual’s personality on the core beliefs in UTAUT2 and AVA. This is pivotal because while the role of personality for shaping AV attitudes and acceptance has been acknowledged, it is still little understood.

Our second major theoretical contribution is the investigation of cross-country differences in the role of the variables in our research model for the acceptance of CondAVs. Considering the specific cultural and social context is pivotal to respond to the needs and preferences of the different European and non-European markets.

A better understanding of the target personalities of potential consumers of AVs is critical to ensure that manufacturers can tailor their products around the personalities of their customers during development and commercialization^7^. Designing AVs around the personality traits of its users may overcome the fear of using AVs, thus promoting trust and AVA^14^, and the shift towards more sustainable mobility^11^.

In the subsequent sections, we will now review the various ways in which personality as measured by the big five can influence attitudes towards AVs and AVA.

### Literature review

#### Openness

Openness has been associated with intellect, culture, intelligence, intellectual interests, and intellectance. Individuals scoring high in openness have a high level of intellectual curiosity, and the tendency to actively seek new and unconventional ideas and experiences. These individuals tend to be curious, inquisitive, imaginative, artistic, more likely to engage in problem-solving^15^, and to use new technology^11^. The effect of an individual’s openness on expectations towards AVs was not significant in^16^. Note that expectations towards AVs was measured by three items on a seven-point Likert rating scale from 1 (low) to 7 (high), i.e. ,‘How would you rate your overall expectations regarding the driving of a self-driving car?’, ‘How would you rate your expectations regarding the effectiveness of a self-driving car?’, and ‘How would you rate your expectations regarding the safety of a self-driving car?’^16^. Openness had a positive effect on individual’s eagerness or decision to adopt AVs^7,17^, and the adoption intention of electric vehicles (EVs)^18^.

Little is known about the indirect effects of openness on the independent variables in our extended UTAUT2 in the field of AVA^19^ extended UTAUT by the big five personality traits to explain technology acceptance in higher education, and did not find support for their theorized positive effect of openness on performance expectancy.

The negative bivariate relationship between openness and trust in automated driving in^20^ suggests that individuals who were more open to new experiences had lower trust in automation, or vice versa. In^17^, openness was negatively related to concerns with AVs, but the effect size was small and not significant. We expect that the effect of openness on driver engagement will be negative because individuals scoring high in openness are more likely to embrace new and novel ideas. Keeping the driver engaged in the driving task may contradict their idea of automated driving, promising a driverless future with drivers becoming passengers who can use the travel time for productive and recreational purposes other than driving.

#### Conscientiousness

Conscientiousness has been associated with competence or efficiency, achievement striving, and self-discipline, and task orientation^15^. Conscientious individuals have a strong tendency to plan ahead, are goal-oriented, and have a strong sense of purpose. They are typically well-organized, structured, dutiful, and persistent^11,15^. Conscientious individuals are more likely to use new technologies that are useful and productive^11^. In^16^, conscientiousness had a positive effect on individual’s expectations towards AVs. Other studies found both negative^17^ and positive^7^ associations with the adoption of AVs. The theorized positive effect of conscientiousness on performance expectancy in higher education could not be supported in^19,21^ found that the positive effect of conscientiousness on the actual use of technology in higher education was significant, whereas conscientiousness did not explain the intention to use the technology.

We posit that conscientious individuals are more likely to consider the driving experience in CondAVs positive as they are more likely to view CondAVs as more useful than human-controlled cars. Therefore, we expect that conscientiousness will have a positive effect on all UTAUT2 independent variables, and trust. The effect of conscientiousness on driver engagement is expected to be negative as keeping the driver engaged in the driving task may mitigate the benefits that conscientious individuals may associate with the use of CondAVs.

#### Extraversion

Extraversion has been associated with confidence, self-expression, assertiveness, and power^15^. It describes an individual’s tendency to actively engage with the social world, and is thus described by activities such as socialability, exuberance, energy, social extraversion, and positive emotion^11,15^. It represents an energetic approach to life and is generally characterized by excitement- or adventure-seeking, and positive emotions^15^. Extraverts are described as action-oriented individuals with a higher willingness to try out new opportunities. In^16^, extraversion had a positive effect on individual’s expectations towards AVs. The effect of extraversion on concerns with AVs was not significant in^17^. Extraversion did explain the intention to adopt EVs^18^, the adoption of AVs^23^, and AVA^21^. Extraversion had a negative effect on actual use of technology in higher education, while the effect on the intention to use the technology was not significant ^19^. The hypothesized effect of extraversion on social influence was also not supported in the field of technology acceptance in higher education^19^. Extraverted people had a higher level of trust in machines, and AVs^21,24^. Informed by the results of these studies, we expect positive effects of extraversion on the independent variables in UTAUT2, and trust, and a negative effect on driver engagement.

#### Agreeableness

Agreeableness has been associated with social adaptability, likeability, friendly compliance, and love^15^. Individuals scoring high in agreeableness like to cooperate with others, and have a strong need for social harmony^11^. It has been associated with a prosocial orientation towards others, compliance, altruism, and with being trustworthy (forgiving)^11,15^. Agreeableness had a positive effect on individual’s expectations towards AVs^23^, whereas it did not affect concerns with AVs in^17^. It was positively related to trust, intention to use AVs, and AVA^21^. Agreeableness had a positive effect on an individual’s willingness to drive and own an AV^26^, whereas the effect of agreeableness on AV adoption intention was negative^7^. The effect of agreeableness on the adoption intention of EVs was positive in^18^, whereas it did not predict the intention to use technology in higher education^22^. Contrary to theoretical assumptions, agreeableness did not affect social influence and facilitating conditions in higher education in^19^. Agreeableness did not influence trust in AVs^27^, while its effect on (dispositional interpersonal) trust in an automated driving system was positive^24^. Despite inconclusive scientific evidence, we expect positive effects of agreeableness on the independent variables in our extended UTAUT2, and trust, and a negative effect on driver engagement.

#### Neuroticism

Neuroticism has been associated with low emotional stability, ego strength (anxiety), dominance-assurance, satisfaction, and affect^15^. Neurotic individuals are more likely to experience constant negative emotions, such as stress, nervousness, anxiety, hopelessness, paranoia, and depression, and negative feelings towards new and unexperienced things. They tend to be more risk-averse, cautious, and skeptical to adopt new technology^11,28^ found small effects of neuroticism on individual’s behavioral intentions to use AVs, which implies that respondents high in neuroticism were less likely to intend to use AVs. Neuroticism did not explain the intention to adopt EVs^18^. In the study of^25^, neurotic respondents were less comfortable with data transmission by AVs, while agreeable respondents were more comfortable. In^17^, emotional stability (low neuroticism) was positively related to an individual’s eagerness to adopt AVs, and negatively related to concerns with AVs. In the study of^16^ neuroticism had a negative effect on trust, and in^20^ neuroticism was negatively related to trust in AVs, and AVA. The hypothesized negative effect of neuroticism on trust in automated driving systems could not be confirmed in^24,21^ found that the effect of neuroticism on actual use was negative, while it did not explain the intention to use technology in higher education^19^ found that neuroticism had a negative effect on performance expectancy, and facilitating conditions. Based on these findings and theoretical reasoning, we expect that neuroticism will have a negative effect on the independent variables in our extended UTAUT2, and trust, and a positive effect on driver engagement.

### Hypotheses development

Based on the above review, we derived the following testable hypotheses, as presented in Table 1. We will not formulate hypotheses that specify the direct main effects of the independent variables performance expectancy, social influence, facilitating conditions, trust and driver engagement on the dependent variable behavioral intention in our extended UTAUT2 model as they were addressed in detail in our previous study^6^.

**Table 1:** Testable hypotheses (H = hypothesis).

H #	Structural path	Expression
H1	Performance expectancy → Behavioral intention	Performance expectancy will have a positive effect on the behavioral intention to use CondAVs, such as that respondents providing high scores for performance expectancy are more likely to provide high scores for behavioral intention.
H2	Facilitating conditions → Behavioral intention	Facilitating conditions will have a positive effect on the behavioral intention to use CondAVs, such as that respondents providing high scores for facilitating conditions are more likely to provide high scores for behavioral intention.
H2	Social influence → Behavioral intention	Social influence will have a positive impact on the behavioral intention to use CondAVs, such as that respondents providing high scores for social influence are more likely to provide high scores for behavioral intention.
H4	Hedonic motivation → Behavioral intention	Hedonic motivation will have a positive effect on the behavioral intention to use CondAVs, such as that respondents providing high scores for hedonic motivation are more likely to provide high scores for behavioral intention.
H4	Trust → Behavioral intention	Trust will have a positive effect on the behavioral intention to use CondAVs, such as that respondents providing high scores for trust in CondAVs are more likely to provide high scores for behavioral intention.
H5	Driver engagement → Behavioral intention	Driver engagement will have a negative effect on the behavioral intention to use CondAVs, such as that respondents providing high scores for driver engagement are less likely to provide high scores for behavioral intention.
H6	Openness → Behavioral intention	Openness to new experiences will have a positive effect on the behavioral intention to use CondAVs, such as that respondents providing high scores for openness are more likely to provide high scores for behavioral intention.
H7	Conscientiousness → Behavioral intention	Conscientiousness will have a positive effect on the behavioral intention to use CondAVs, such as that respondents providing high scores for conscientiousness are more likely to provide high scores for behavioral intention.
H8	Extraversion → Behavioral intention	Extraversion will have a positive effect on the behavioral intention to use CondAVs, such as that respondents providing high scores for extraversion are more likely to provide high scores for behavioral intention.
H9	Agreeableness → Behavioral intention	Agreeableness will have a positive effect on the behavioral intention to use CondAVs, such as that respondents providing high scores for agreeableness are more likely to provide high scores for behavioral intention.
H10	Neuroticism → Behavioral intention	Neuroticism will have a negative impact on the behavioral intention to use CondAVs, such as that respondents providing high scores for neuroticism are less likely to provide high scores for behavioral intention.
H6a	Openness → Performance expectancy	Openness will have a positive effect on performance expectancy, such as that respondents providing high scores for openness are more likely to provide high scores for performance expectancy.
H6b	Openness → Social influence	Openness will have positive effect on social influence, such as that respondents providing high scores for openness are more likely to provide high scores for social influence.
H6c	Openness → Facilitating conditions	Openness will have a positive effect on facilitating conditions in CondAVs, such as that respondents providing high scores for openness are more likely to provide high scores for facilitating conditions.
H6d	Openness → Driver engagement	Openness will have a negative effect on driver engagement in CondAVs, such as that respondents providing high scores for openness are less likely to provide high scores for driver engagement.
H6e	Openness → Trust	Openness will have a positive effect on trust in CondAVs, such as that respondents providing high scores for openness are more likely to provide high scores for trust.
H7a	Conscientiousness → Performance expectancy	Conscientiousness will have a positive effect on performance expectancy, such as that respondents providing high scores for conscientiousness are more likely to provide high scores for performance expectancy.
H7b	Conscientiousness → Social influence	Conscientiousness will have a positive effect on social influence, such as that respondents providing high scores for conscientiousness are more likely to provide high scores for social influence.
H7c	Conscientiousness → Facilitating conditions	Conscientiousness will have a positive effect on facilitating conditions in CondAVs, such as that respondents providing high scores for conscientiousness are more likely to provide high scores for facilitating conditions.
H7d	Conscientiousness → Driver engagement	Conscientiousness will have a negative effect on driver engagement in CondAVs, such as that respondents providing high scores for conscientiousness are less likely to provide high scores for driver engagement.
H7e	Conscientiousness → Trust	Conscientiousness will have a positive effect on trust in CondAVs, such as that respondents providing high scores for conscientiousness are more likely to provide high scores for trust.
H8a	Extraversion → Performance expectancy	Extraversion will have a positive effect on performance expectancy, such as that respondents providing high scores for extraversion are more likely to provide high scores for performance expectancy.
H8b	Extraversion → Social influence	Extraversion will have a positive effect on social influence, such as that respondents providing high scores for extraversion are more likely to provide high scores for social influence.
H8c	Extraversion → Facilitating conditions	Extraversion will have a positive effect on facilitating conditions in CondAVs, such as that respondents providing high scores for extraversion are more likely to provide high scores for facilitating conditions.
H8d	Extraversion → Driver engagement	Extraversion will have a negative effect on driver engagement in CondAVs, such as that respondents providing high scores for extraversion are less likely to provide high scores for driver engagement.
H8e	Extraversion → Trust	Extraversion will have a positive effect on trust in CondAVs, such as that respondents providing high scores for extraversion are more likely to provide high scores for trust.
H9a	Agreeableness → Performance expectancy	Agreeableness will have a positive effect on performance expectancy, such as that respondents providing high scores for agreeableness are more likely to provide high scores for performance expectancy.
H9b	Agreeableness → Social influence	Agreeableness will have a positive effect on social influence, such as that respondents providing high scores for agreeableness are more likely to provide high scores for social influence.
H9c	Agreeableness → Facilitating conditions	Agreeableness will have a positive effect on facilitating conditions in CondAVs, such as that respondents providing high scores for agreeableness are more likely to provide high scores for facilitating conditions.
H9d	Agreeableness → Driver engagement	Agreeableness will have a negative effect on driver engagement in CondAVs, such as that respondents providing high scores for driver engagement are less likely to provide high scores for driver engagement.
H9e	Agreeableness → Trust	Agreeableness will have a positive effect on trust in CondAVs, such as that respondents providing high scores for agreeableness are more likely to provide high scores for trust.
H10a	Neuroticism → Performance expectancy	Neuroticism will have a negative effect on performance expectancy, such as that respondents providing high scores for neuroticism are less likely to provide high scores for performance expectancy.
H10b	Neuroticism → Social influence	Neuroticism will have a negative effect on social influence, such as that respondents providing high scores for neuroticism are less likely to provide high scores for social influence.
H10c	Neuroticism → Facilitating conditions	Neuroticism will have a negative effect on facilitating conditions in CondAVs, such as that respondents providing high scores for neuroticism are less likely to provide high scores for facilitating conditions
H10d	Neuroticism → Driver engagement	Neuroticism will have a positive effect on driver engagement in CondAVs, such as that respondents providing high scores for neuroticism are more likely to provide high scores for driver engagement.
H10e	Neuroticism → Trust	Neuroticism will have a negative effect on trust in CondAVs, such as that respondents providing high scores for neuroticism are less likely to provide high scores for trust.

## Methodology

### Procedure

An online questionnaire was developed in the L3Pilot project (www.l3pilot.eu), and distributed to a sample in Brazil, China, France, Germany, Hungary, Japan, Russia, U.K., and the U.S. that is representative of its national country population in terms of age, gender, and income, respectively. The selection of the countries was based on their current and future car market size, geographical representation and leadership in the development of automated driving technology^29^. To recruit car drivers, respondents were excluded from the questionnaire if they indicated that they never make use of private, carsharing, and rental cars as driver^2^.

The implementation of the questionnaire was conducted by the German market research institute INNOFACT AG (www.innofact.com*)* using the questionnaire tool EXAVO (https://www.exavo.de/surveytainment/*).* INNOFACT AG hired an official translation bureau, which translated the questionnaires into the different national languages of the countries in which the questionnaires were administered. INNOFACT AG sent the invitations to participate in the questionnaire via email to its online panels. Once a representative sample in each country was obtained, participation in the questionnaire was closed. To enhance data quality, INNOFACT AG used several technologies, such as ensuring that only humans and no bots with suspect proxies or email addresses, could complete the questionnaire. Moreover, respondents were not allowed to take the questionnaire more than once, e.g., via multiple email or panel accounts from the same computer.

Respondents were informed that the questionnaire was executed as part of the L3Pilot project, and that their responses may be analysed and / or published for research purposes. A link to the project was provided so that respondents could obtain more information about the project if needed. We also mentioned that it would take around 20 min to complete the survey, and that responses would be treated anonymously. We did not seek approval for this study from the ethics committee as no ethics application had to be submitted for this study as the study-related data processing involves anonymous data. For this reason, regulations on the protection of personal data are not relevant in the context of this study nor are other ethical concerns affected. Moreover, when respondents were invited to the study, they were informed that their participation in the questionnaire is voluntary, that they could withdraw from the questionnaire at any time, and withdraw their responses to questions they did not want to answer. Respondents were financially compensated for their participation in the questionnaire. Respondents from Germany received 1€ for completing the questionnaire. Respondents from the remaining countries received vouchers worth between 0.80‒1.00€ per respondent.

### Instrument

The questionnaire was divided into the Sections A‒F.

Section A presented questions about respondents’ personal information, including their age, gender, highest level of education completed, number of children aged younger than 19 years per household, access to a valid driver’s license, and annual driving mileage.

Section B is about respondents’ personality.

In Section C, respondents were asked questions about their awareness of AVs, and how often they read / watch / listen to information about AVs.

In Section D, respondents were asked to indicate their agreement with questions representing the UTAUT2 questions, trust, driver engagement, and secondary task engagement.

After this section, the sample was randomly split into two equal streams, maintaining the original age and gender distribution.

Section E presents respondents with several questions to examine their attitude towards different Automated Driving Functionalities (ADFs).

Finally, Section F asked respondents to provide their responses to questions representing their attitudes towards driving, and experiences with driver assistance systems.

### Data analysis

We applied structural equation modeling in two main steps.

In the first step of the analysis, a confirmatory factor analysis was conducted to estimate the measurement relations between the latent constructs and underlying questionnaire items by assessing the internal consistency reliability (i.e., Cronbach’s alpha), composite reliability, convergent validity, and discriminant validity. To assess convergent validity, the factor loadings (i.e., lambda’s) should be significant, exceeding the threshold of 0.60 on their respective scales. The Average Variance Extracted (AVE) should exceed the threshold of 0.50, and the construct reliability (CR), and 4) Cronbach’s alpha values should be higher than 0.60. Discriminant validity (i.e., uni-dimensionality) of the latent constructs is established if the square root of the AVE of each latent construct exceeds the correlation coefficients between the latent constructs^30,31^.

We also tested for measurement invariance across countries to assess psychometric equivalence, i.e., the extent to which a question has the same relationship to an underlying latent construct across countries. The assessment of measurement invariance was conducted in several steps, imposing more rigid requirements on the psychometric properties with every step. In the first step, configural invariance assesses whether the basic measurement model is invariant across countries. Metric invariance assesses to what extent the factor loadings are comparable. This step assesses whether each observed variable has the same relationship to their underlying latent variable. Scalar invariance (strong invariance) applies if the factor loadings and intercepts are comparable across countries. This implies that the observed variables have the same expected values for people with the same level of the latent variable. Invariant error terms (strict measurement invariance) applies if factor loadings, intercepts, and error variances are invariant across countries^32^. The fit of the model is considered acceptable if the Comparative Fit Index (CFI) ≥ 0.95, Root Mean Square Error of Approximation (RMSEA) ≤ 0.08, and the Standardized Root Mean Square Residual (SRMR) ≤ 0.06^31^.

In the second step of the analysis, a structural equation modeling analysis was run, which is based on the acceptable measurement model identified in the first step of the analysis. This involves testing the structural path relationships between the latent constructs in the model, examining the standardized regression coefficients, standard error terms, significance levels, and variance accounted for in the variables. We used Maximum Likelihood Estimation (MLE) for this estimation. With 9,339 responses, our study fulfills the “ten times rule of thumb” which suggests a sample size of ten times the maximum number of independent variables in the structural equation model^32^, p. 325].

The analysis was performed in R. The code that was run for the estimation of the measurement and structural equation model is provided as supplementary material.

## Results

### Respondents

Responses were collected between February 3rd and 17th, 2021. We applied strict data filtering to enhance data quality. This included the removal of respondents whose time to complete the questionnaire was 33% below the median length, and who provided the same answer (i.e., strongly disagree, neutral, agree strongly) to the questions q14r.1‒q14r.20. We retained 9,339 valid responses for the analysis.

The mean age of respondents is 41.51 (in years) (SD = 13.84). The binary sample is gender-balanced, with 48% of respondents being male, and 52% being female. 29% of respondents completed their college degree (no finished studies), followed by 25% of respondents completing a college degree, 13% had a high school diploma with apprenticeship / professional training, and 3% had a high school diploma without apprenticeship / professional training. The majority of respondents (52%) had no children aged younger than 19 years, followed by 28% having 1 child, 16% having 2 children, and 4% having 3 children. 23% of respondents indicated to drive between 3.000 and 6.000 miles and 9.000–12.000 miles per year, respectively, followed by 19% of respondents who reported to drive between 1.000 and 3.000 and 6.000–9.000 miles annually, respectively. 14% drove less than 1.000 miles annually, and 3% drove between 12.000 and 30.000 and more than > 30.000 miles annually. 

### Confirmatory factor analysis

The results of the confirmatory factor analysis have shown that the standardized factor loadings are $\:\ge\:$ 0.60 for all constructs after removing the questionnaire items with loadings that did not meet this threshold. After omitting these items from the measurement model, facilitating conditions, hedonic motivation and the personality constructs were single-item constructs. As single-item constructs, their psychometric properties couldn’t be computed. The use of single-item constructs in structural equation models is acceptable if their use is limited, and justified^31^. The validity and reliability of the items representing facilitating conditions and hedonic motivation was supported in our previous study on the acceptance of CondAVs^2^. Cronbach’s alpha and composite reliability are higher than the common threshold of 0.60 for all constructs except for driver engagement. This demonstrates that the constructs are largely internally consistent (internal consistency reliability). The Average Variance Extracted (AVE) exceed the recommended threshold of $\:\ge\:\:$0.50 for all latent constructs. The fit of the measurement model is acceptable, with the indexes exceeding the recommended thresholds (CFI = 0.98 (≥ 0.95), RMSEA = 0.05 (≤ 0.08), SRMR = 0.02 (≤ 0.06). The x^2^ test statistic (x^2^/df, degrees of freedom) is 23.52, exceeding the recommended threshold of 2.5. The x^2^ test statistic is sensitive to sample size, with larger sample sizes leading to higher values^13,30^.

Table 2 presents the results of the confirmatory factor analysis.

**Table 2:** Confirmatory factor analysis results (ƛ = lambda, ⍺ = Cronbach’s alpha, CR = composite reliability, AVE = average variance extracted).

Latent variable	Observed variable	ƛ	⍺	CR	AVE
Performance expectancy (PE)	PE1: Using a conditionally automated car would help me reach my destination more safely (q14r.1)	0.82	0.81	0.85	0.68
	PE2: Using a conditionally automated car would help me reach my destination more comfortably (q14r.2)	0.83			
Facilitating conditions (FC)	FC1: I could acquire the necessary knowledge to use a conditionally automated car (q14r.17)	1.00	–	–	–
Social influence (SI)	SI1: Using a conditionally automated car would give me status and prestige among people important to me (q14r.12)	0.74	0.79	0.82	0.66
	SI2: It would make me proud to own a conditionally automated car (q14r.13)	0.89			
Hedonic motivation (HM)	HM1: Using a conditionally automated car would be enjoyable (q14r.14)	1.00	–	–	–
Trust (TRU)	TRU1: I would be suspicious of conditionally automated cars (reverse-scaled) (q12.1)	0.82	0.81	0.80	0.68
	TRU2: I would feel hesitant about using a conditionally automated car (reverse-scaled) (q12.4)	0.83			
Driver engagement (DE)	DE1: I would not want to monitor what the conditionally automated car is doing when it is in control (reverse-scaled) (q14r.7)	0.70	0.66	0.67	0.50
	DE2: I would not want to stop the other activity I am doing to respond to requests from the car to take over control (reverse-scaled) (q14r.8)	0.71			
Big five personality traits					
Openness (O)	O1. I see myself as someone who has few artistic interests (q8.5)	–	–	–	–
	O2. I see myself as someone who has an active imagination (q8.10)	1.00	–	–	–
Conscientiousness (C)	C1: I see myself as someone who tends to be lazy (q8.3)	–	–	–	–
	C2. I see myself as someone who does a thorough job (q8.8)	1.00	–	–	–
Extraversion (E)	E1: I see myself as someone who is reserved (q8.3)	–	–	–	–
	E2. I see myself as someone who is outgoing, sociable (q8.6)	1.00	–	–	–
Agreeableness (A)	A1. I see myself as someone who is generally trusting (q8.2)	1.00	–	–	–
	A2: I see myself as someone who tends to find fault with others (q8.7)	–	–	–	–
Neuroticism (N)	N1: I see myself as someone who handles stress well (q8.4)	–	–	–	–
	N2. I see myself as someone who gets nervous easily (q8.9)	1.00	–	–	–
Behavioral intention (BI)	BI1: I plan to use a conditionally automated car once it becomes available (q12.5)	0.83	0.85	0.90	0.80
	BI2: I intend to use a conditionally automated car in the future (q15.1)	0.89			

As shown by Table 3, the square root of the AVE of all constructs exceed the correlation coefficients of all constructs, demonstrating that the latent constructs are sufficiently distinct (discriminant validity).

**Table 3:** Discriminant validity test; spearman-rank inter-construct correlation matrix.

Construct	PE	SI	FC	HM	DE	TRU	O	C	E	A	*N*	BI
PE	0.82											
**SI**	0.65 ***	**0.81**										
**FC**	0.44 ***	0.36 ***	**1**									
**HM**	0.73 ***	0.69 ***	0.45 ***	**1**								
**DE**	-0.31 ***	-0.41 ***	-0.03 *	-0.30 ***	**0.71**							
**TRU**	0.49 ***	0.40 ***	0.26 ***	0.51 ***	-0.12 ***	**0.82**						
**O**	0.18 ***	0.18 ***	0.21 ***	0.18 ***	-0.08 ***	0.05 ***	**1**					
**C**	-0.01	0.01	0.10 ***	0.00	0.03 ***	-0.12 ***	0.20 ***	**1**				
**E**	0.19 ***	0.21 ***	0.14 ***	0.19 ***	-0.10 ***	0.08 ***	0.27 ***	0.27 ***	**1**			
**A**	0.15 ***	0.16 ***	0.17 ***	0.15 ***	-0.06 ***	0.02 ***	0.19 ***	0.14 ***	0.32 ***	**1**		
**N**	0.03 ***	0.05 ***	-0.06 ***	0.01	-0.15 ***	-0.19 ***	0.01	-0.07 ***	-0.21 ***	-0.07 ***	**1**	
**BI**	0.70 ***	0.69 ***	0.40 ***	0.72 ***	-0.36 ***	0.57 ***	0.19 ***	-0.03 ***	0.19 ***	0.14 ***	0.02	**0.89**

Note: *p < 0.05; **p < 0.01; ***p < 0.001, all remaining correlations are not significant.

PE = Performance Expectancy, SI = Social Influence, DE = Driver Engagement, TRU = Trust, O = Openness, C = Conscientiousness, E = Extraversion, A = Agreeableness, N = Neuroticism, BI = Behavioral Intention.

### Structural equation modeling analysis

Tables 4 and 5 present the results of the structural equation modeling analysis, examining the direct and indirect effects of the variables in the extended UTAUT2 and the five personality traits on the behavioral intention to use CondAVs across and between countries. All five personality traits, except for conscientiousness, predict the behavioral intention to use CondAVs, but the effect sizes are small (< 0.10). Of all the predictors, social influence is the strongest predictor of the behavioral intention to use CondAVs.

To estimate the between-country effects, we run separate structural equation models, as presented in in the supplementary material 3. We found significant differences between countries, with large differences in the effect sizes, and significance levels. The cross-country and between-country analysis has shown that most of the personality traits are not strong predictors of the behavioral intention to use CondAVs.

The results will be discussed in detail in the subsequent sections.

**Table 4:** Cross-country analysis predicting behavioral intention to use CondAVs.

Hypotheses		
Predicting Behavioral Intention	*R*^2^ in BI	0.72
H1	Performance Expectancy → Behavioral Intention	0.34***
H2	Facilitating Conditions → Behavioral Intention	0.08***
H3	Social Influence → Behavioral Intention	0.46***
H4	Hedonic Motivation → Behavioral Intention	0.31***
H5	Trust → Behavioral Intention	0.39***
H6	Driver Engagement → Behavioral Intention	-0.17***
**Predicting Performance Expectancy**	***R*** ^**2**^ **in Performance Expectancy**	**0.08**
H6a	Openness → Performance Expectancy	0.16***
H7a	Consciousness → Performance Expectancy	-0.08***
H8a	Extraversion → Performance Expectancy	0.13***
H9a	Agreeableness → Performance Expectancy	0.11***
H10a	Neuroticism → Performance Expectancy	0.07***
**Predicting Facilitating Conditions**	***R*** ^***2***^ **in Facilitating Conditions**	**0.07**
H6b	Openness → Facilitating Conditions	0.17***
H7b	Consciousness → Facilitating Conditions	0.04***
H8d	Extraversion → Facilitating Conditions	0.04***
H9d	Agreeablenes → Facilitating Conditions	0.11***
H10d	Neuroticism → Facilitating Conditions	-0.05***
**Predicting Social Influence**	***R*** ^***2***^ **in Social Influence**	**0.10**
H6c	Openness → Social Influence	0.15***
H7c	Consciousness → Social Influence	-0.10***
H8c	Extraversion → Social Influence	0.20***
H9c	Agreeableness → Social Influence	0.11***
H10c	Neuroticism → Social Influence	0.09***
**Predicting Hedonic Motivation**	**R** ^**2**^ **in Hedonic Motivation**	**0.07**
H6d	Openness → Hedonic Motivation	0.14***
H7d	Consciousness → Hedonic Motivation	-0.08***
H8d	Extraversion → Hedonic Motivation	0.15***
H9d	Agreeableness → Hedonic Motivation	0.09***
H10e	Neuroticism → Hedonic Motivation	0.04***
**Predicting Trust**	**R** ^**2**^ **in Trust**	**0.07**
H6e	Openness → Trust	0.08***
H7e	Consciousness → Trust	-0.17***
H8e	Extraversion → Trust	0.06***
H9e	Agreeableness → Trust	0.00
H10e	Neuroticism → Trust	-0.20***
**Predicting Driver Engagement**	***R*** ^***2***^ **in Driver Engagement**	**0.08**
H6f	Openness → Driver Engagement	-0.06***
H7f	Consciousness → Driver Engagement	0.08***
H8f	Extraversion → Driver Engagement	-0.17***
H9f	Agreeableness → Driver Engagement	-0.03*
H10f	Neuroticism → Driver Engagement	-0.23***

*Note: R*^2^ is the variance accounted for in the predicted variable. Presented next to *R*^2^ are the standardized beta coefficients *β*, and the significance levels *p < 0.05; **p < 0.01; ***p < 0.001. No significance level indicates that the relationship is not significant.

**Table 5:** Between-country analysis predicting behavioral intention to use CondAVs.

Hypothethical Path		U.S.	UK	FR	HU	DE	CN	BR	JP	RU
Predicting Behavioral Intention (BI)		0.81	0.73	0.77	0.82	0.67	0.81	0.78	0.63	0.64
H6a	PE → BI	0.38 ***	0.38 ***	0.32 ***	0.29 ***	0.19 ***	0.47 ***	0.42 ***	0.30 ***	0.43 ***
H7a	TRU → BI	0.23 ***	0.44 ***	0.41 ***	0.69 ***	0.53 ***	0.21 ***	0.39 ***	0.51 ***	0.44 ***
H8a	SI → BI	0.54 ***	0.40 ***	0.50 ***	0.43 ***	0.41 ***	0.26 ***	0.47 ***	0.40 ***	0.31 ***
H9a	FC → BI	0.07 **	0.13 ***	0.10 ***	0.10 ***	0.05 *	0.10 ***	0.04	0.10 ***	0.12 ***
H10a	HM→ BI	0.35 ***	0.31 ***	0.26 ***	0.21 ***	0.42 ***	0.17 ***	0.24 ***	0.22 ***	0.30 ***
H11a	DE→ BI	-0.08 **	-0.09 **	-0.17 ***	-0.19 ***	-0.07	-0.33 ***	-0.08 *	-0.01	-0.02
**Predicting Performance Expectancy (PE)**		**0.12**	**0.10**	**0.12**	**0.03**	**0.01**	**0.36**	**0.12**	**0.05**	**0.05**
H6b	O → PE	0.17 ***	0.08 *	0.24 ***	0.10 **	0.07 *	0.27 ***	0.11 ***	0.15 ***	0.03
H7b	C → PE	-0.07*	-0.04	0.00	0.07	-0.01	0.23 ***	0.12 ***	0.04	0.07
H8b	E → PE	0.15 ***	0.22 ***	0.04	-0.04	0.06	0.23 ***	0.16 ***	0.07	0.05
H9b	A → PE	0.12 **	0.09 *	0.17 ***	0.08 *	0.00	0.08 *	0.19 ***	0.02	0.15 ***
H10b	N → PE	0.16 ***	0.21 ***	-0.02	0.06	0.04	-0.02	-0.05	0.11 **	-0.01
**Predicting Social Influence (SI)**		**0.19**	**0.13**	**0.11**	**0.03**	**0.03**	**0.37**	**0.12**	**0.08**	**0.05**
H6c	O → SI	0.23 ***	0.05	0.22 ***	0.10 **	-0.02	0.20 ***	0.13 ***	0.18 ***	0.07 *
H7c	C→ SI	-0.08 *	-0.12 ***	-0.04	-0.01	0.00	0.15 ***	0.07 *	0.02	0.06
H8c	E→ SI	0.19 ***	0.28 ***	0.09 *	0.03	0.14 ***	0.37 ***	0.19 ***	0.14 ***	0.07
H9c	A→ SI	0.16 ***	0.11 **	0.17 ***	0.08 *	0.04	0.11 **	0.18 ***	0.04	0.12 **
H10c	N→ SI	0.18 ***	0.22 ***	0.05	0.08 *	0.12 ***	0.06	-0.02	0.09 *	0.00
**Predicting Facilitating Conditions (FC)**		**0.08**	**0.04**	**0.07**	**0.04**	**0.04**	**0.16**	**0.09**	**0.06**	**0.10**
H6d	O → FC	0.17 ***	0.10 **	0.16 ***	0.11 ***	0.14 ***	0.22 ***	0.10 ***	0.15 ***	0.06 *
H7d	C → FC	0.11 ***	0.11 ***	0.10 **	0.14 ***	0.05	0.13 ***	0.07 *	0.10 **	0.14 ***
H8d	E → FC	0.06	0.04	0.05	-0.01	-0.05	0.09 *	0.06 *	0.03	0.05
H9d	A → FC	0.09**	0.05	0.07*	-0.01	0.08*	0.11 ***	0.23 ***	0.03	0.15 ***
H10d	N → FC	0.04	0.00	-0.03	-0.02	-0.06*	-0.10 ***	-0.06	0.09 **	-0.05
**Predicting Hedonic Motivation (HM)**		**0.12**	**0.08**	**0.06**	**0.01**	**0.01**	**0.17**	**0.08**	**0.08**	**0.04**
H6e	O → HM	0.21 ***	0.09 **	0.15 ***	0.06*	0.07 *	0.18 ***	0.12 ***	0.18 ***	0.04
H7e	C → HM	-0.05	-0.08 *	-0.01	0.02	-0.04	0.14 ***	0.03	0.03	0.07 *
H8e	E → HM	0.12 ***	0.20 ***	0.09 **	0.02	0.05	0.16 ***	0.12 ***	0.12 ***	0.06
H9e	A → HM	0.13 ***	0.08 *	0.11 ***	0.03	0.04	0.10 ***	0.16 ***	0.05	0.10 **
H10e	N → HM	0.14 ***	0.15 ***	0.03	0.04	0.03	-0.05	-0.06 *	0.07 *	-0.03
**Predicting Trust (TRU)**		**0.06**	**0.03**	**0.07**	**0.02**	**0.02**	**0.37**	**0.11**	**0.03**	**0.02**
H6f	O → TRU	0.08 *	-0.03	0.07 *	-0.03	0.03	0.09 **	0.10 **	0.07	0.05
H7f	C → TRU	-0.16 ***	-0.12 ***	-0.10 **	-0.08 *	-0.06	0.05	0.04	-0.10	0.00
H8f	E → TRU	0.01	0.09 *	-0.04	-0.04	-0.01	-0.03	0.10 **	0.05	0.05
H9f	A → TRU	0.05	-0.02	0.14 ***	-0.04	-0.01	-0.10 **	0.09 **	-0.03	-0.01
H10f	N → TRU	-0.20 ***	-0.10 **	-0.20 ***	-0.08 *	-0.14 ***	-0.57 ***	-0.24 ***	-0.12 ***	-0.13 ***
**Predicting Driver Engagement (DE)**		**0.15**	**0.15**	**0.09**	**0.06**	**0.05**	**0.35**	**0.05**	**0.02**	**0.01**
H6f	O → DE	-0.09 *	-0.02	-0.17 ***	-0.05	0.06	-0.08	-0.04	-0.07	-0.03
H7f	C → DE	0.13 ***	0.20 ***	0.20 ***	0.11 **	0.15 **	-0.06	0.03	0.09 *	-0.10 *
H8f	E → DE	-0.20 ***	-0.28 ***	-0.05	-0.03	-0.12 *	-0.37 ***	-0.05	-0.12 *	-0.03
H9f	A → DE	-0.11 **	-0.04	-0.12 **	-0.16 ***	-0.02	-0.14 ***	0.03	-0.04	0.03
H10f	N → DE	-0.27 ***	-0.29 ***	-0.13 ***	-0.16 ***	-0.12 *	-0.37 ***	-0.22 ***	-0.02	-0.05

*Note: R*^2^ is the variance accounted for in the predicted variable. Presented next to *R*^2^ are the standardized beta coefficients *β*, and the significance levels *p < 0.05; **p < 0.01; ***p < 0.001. No significance level indicates a non-significant relationship.

## Discussion

We extended prior research on AVA by examining how an individual’s personality affects general attitudes and acceptance of CondAVs. The role of personality for the acceptance and use of technology has been established in other domains. However, its role for the acceptance and use of CondAVs is still little understood. We could not identify other studies examining the effect of the personality traits on attitudes and acceptance of CondAVs. UTAUT2 adjusted to the context of CondAVs was empirically tested using questionnaire data from 9,339 car drivers from European and non-European markets. We investigated both the direct and indirect effects of an individual’s personality on the UTAUT2 constructs across countries, and between countries. The analysis has provided strong support for our adjusted UTAUT2 model. Most of our hypotheses were supported. Nevertheless, most of the effects of the independent variables of the extended UTAUT2 and the personality traits on behavioral intention were relatively small. The between-country analysis of the direct and indirect effects has shown some notable differences in the effect sizes of the independent variables in our model.

Social influence was the strongest predictor of the behavioral intention to use CondAVs, followed by trust and performance expectancy. In^16^, the effect of trust on the behavioral intention to use AVs was strongest, followed by social influence, and performance expectancy. The positive effect of social influence on the behavioral intention to use CondAVs was strongest in the U.S., and weakest in China, and Russia. The positive effect of social influence on behavioral intention in France corresponds with^4^. The effect of trust on behavioral intention varied between countries, with the strongest effect in Hungary and the weakest in the U.S. In^33^, the component ‘AV fear’ had a stronger influence on AVA in Japan than in the UK and Germany where the difference between these two countries was not significant.

Performance expectancy had the strongest effect in Russia and the weakest in Germany. A previous study revealed a strong influence of performance expectancy on the behavioral intention to use AVs^4^. In^33^, respondents from Japan provided higher scores for the importance of convenience as factor influencing AVA, followed by happiness and social influence, with respondents in the UK and Japan rating the happiness and social issues as more important than convenience. The effect of the component ‘AV expectations’ on the behavioral intention to use AVs was stronger in the UK than in Germany and Japan, which is in line with our study.

Facilitating conditions and hedonic motivation were the weakest predictors of the behavioral intention across countries. The effect of facilitating conditions was only significant in the UK, France, and Hungary with very small effect sizes. In previous studies, the effect of facilitating conditions on behavioral intention was positive with or without experience^34^, or not significant^4^. The effect of hedonic motivation was strongest in Germany. Hedonic motivation was the strongest predictor of the behavioral intention to use CondAVs in our previous study^2^, and other studies examining user acceptance of AVs^35^.

The negative effect of driver engagement was significant in most countries, and strongest in China. A negative effect size implies that the preference to stay engaged in the driving task negatively influences the behavioral intention to use CondAVs. We could not identify other studies examining this relationship.

### Personality traits

Four out of five personality traits (i.e., openness, extraversion, agreeableness, neuroticism) had positive, yet small, effects on behavioral intention. Our finding that neuroticism had a small but positive effect on behavioral intention corresponds with the study of^36^ which revealed that emotional stability (the opposite of neuroticism) predicted the behavioral intention to use software, but it contrasts the findings in^16^ where no effect on the behavioral intention to use AVs was found. Our finding that openness predicted the behavioral intention to use CondAVs corresponds with^37^ who found that personal innovativeness influenced the behavioral intention to use products containing artificial intelligence. However, it is not in line with the studies of^7,16,38^, which revealed that an innovative personality and openness to experiences did not predict the public acceptance of EVs and of AVs. In^7^, the effect of agreeableness on the intention to adopt AVs was not significant, whereas we found a small positive effect. In^36^, the effect of extraversion on the behavioral intention to use software was not significant, whereas we found a small positive effect of extraversion on behavioral intention to use CondAVs. Our finding that conscientiousness did not predict behavioral intention corresponds with^36^ predicting the behavioral intention to use software by an individual’s personality. In^7^ the effect of conscientiousness on the adoption decision of AVs was positive yet small.

As mentioned before, the effects of the personality traits on the variables in the extended UTAUT2 were relatively small. The strongest positive relationship was found between extraversion and trust in Russia. We also revealed a moderate negative effect of neuroticism on trust in China, suggesting that the Chinese neurotic individuals were less likely to trust CondAVs. In^16^, the effect of neuroticism on trust was negative, and agreeableness had a small positive effect on trust, while the effects of openness, conscientiousness, and extraversion on trust were not significant.

### Scientific and practical contributions

Contrary to widely held beliefs that personality is a critical factor shaping attitudes and acceptance, our study provides some contradictory evidence. The relatively small effect sizes of the personality traits on the independent UTAUT2 variables and behavioral intention suggests that personality may not be a strong predictor of individuals’ beliefs about CondAVs and their acceptance of CondAVs. However, it is also plausible that personality traits other than the big five are more relevant for the prediction of the acceptance of CondAVs, such as an individual’s attachment style [see^10^ for an overview]. In^39^ high sensation seekers rated the aggressive driving style of the AV as natural, whereas this was not found for the low sensation seekers.

Our study has revealed some notable between-country differences in the effect of the personality traits on the independent variables in our model. Neuroticism had the strongest negative effect on driver engagement and trust. Neurotic Chinese respondents had the lowest likelihood to prefer to stay engaged in the driving task when conditionally automated driving was engaged. The effect on trust was consistently negative in all countries, with the strongest effect in Brazil. Given this strong negative effect of neuroticism on trust, car manufacturers and designers should accommodate the development and design of their CondAVs around the needs of this specific neurotic user group [see^7^]. Vehicle characteristics should be identified and advertising strategies and campaigns developed that support the development of trust in CondAVs among neurotic individuals. Given that the effect was strongest in Brazil, catering for the needs of neurotic Brazilian respondents may be effective to promote their trust in CondAVs. None of the five personality traits predicted the perceived enjoyment (hedonic motivation) among the Hungarian respondents, meaning that personality may be disregarded for influencing hedonic motivation among the Hungarians.

Given that the effect of trust on the behavioral intention to use CondAVs was strongest in Hungary, promoting trust in CondAVs among the Hungarian respondents can be particularly effective. The small effect size in the U.S., on the contrary, suggests that promoting trust among the respondents in the U.S. may be less effective to promote acceptance of CondAVs. Given the large effect size of performance expectancy on behavioral intention in China, promoting the perceived benefits of CondAVs in China seems to be an effective way to promote acceptance, whereas this strategy seems to be less promising in Germany where performance expectancy was not a driver of acceptance.

### Limitations and future research

Our study has several limitations.

First, as respondents have not physically experienced CondAVs, hypothetical bias may be present. Hypothetical refers to the discrepancy between revealed and stated preferences^40^. Thus, it is plausible that respondents may have incorrect expectations of the capabilities and limitations of CondAVs, and the user experience, leading to biased estimates. Future research should assess to what extent the stated preferences deviate from the revealed preferences in the field of AVA.

The second limitation pertains to the measurement of the core constructs by the highest-loading questions underlying each construct. Some questions measuring the key UTAUT2 constructs were removed from the analysis as their loading on their underlying construct was not strong enough. Even though we hired a professional translation agency to translate the items into their respective national languages, the meaning of these items across countries may still differ. Each of the five personality traits was represented by a single item in the structural equation modeling analysis as the loadings of the second item was not strong enough to be included as valid and reliable indicator of their underlying latent construct. While this approach was adopted before^35^, it can lead to undermining some facets of the constructs, diminishing content validity. Future research should perform studies for scale development and validation. In line with^35^, we propose to develop and validate additional questions for behavioral intention as the questions representing behavioral intention tend to be generic.

Third, we did not investigate the effect of effort expectancy on behavioral intention given ambiguous scientific evidence, with some studies reporting positive^41^, or no e﻿ffects^42,43^. ^37^ omitted the construct facilitating conditions from their acceptance model due to its controversial role in predicting behavioral intention in studies recruiting respondents without sufficient technology experience. It is plausible that the effect of effort expectancy on behavioral intention is captured by performance expectancy or facilitating conditions due to the strong semantic similarity between these constructs. Future research should investigate the impact of effort expectancy on behavioral intention interacting with performance expectancy and facilitating conditions before and after experience.

Fourth, with regards to the effect of an individual’s personality, our study has limited its focus to one of the most influential personality measures. However, personality is a multi-faceted construct, and other important constructs as captured by the Dark Triad personality traits may be pivotal as well^44^. We recommend future research to examine the effect of the Dark Triad personality traits (i.e., Machiavellianism, Narcissism, Psychopathy)^45^ on user acceptance of AVs, testing the hypothesis that user acceptance will be higher among people scoring higher on the Dark Triad personality traits, considering that AVs will cause fatalities and injuries among road users.

## Electronic supplementary material

Below is the link to the electronic supplementary material.

Supplementary Material 1

Supplementary Material 2

Supplementary Material 3

## Data Availability

All data generated or analysed during this study are included in this published article.
