# Supplementary material for: Examining the effect of personality on user acceptance of conditionally automated vehicles
Source: Sci Rep. 2025 Jan 7;15:1091. doi: 10.1038/s41598-024-84776-4 (PMC11706942; doi:10.1038/s41598-024-84776-4)
Supplement: Supplementary file 3 — Supplementary Material 3 [file 41598_2024_84776_MOESM3_ESM.docx]

1. **Appendix**

*Table A1.* Cross-country analysis predicting behavioral intention to use CondAVs, direct effects

| **Hypothetical path** | ***R^2^* in BI** |
| --- | --- |
|  | **0.86** |
| H1: PE 🡪 BI | 0.23*** |
| H2: FC 🡪 BI | 0.04*** |
| H3: SI 🡪 BI | 0.36*** |
| H4: HM 🡪 BI | 0.08*** |
| H5: TRU 🡪 BI | 0.29*** |
| H6: DE 🡪 BI | -0.09*** |
| H7: O 🡪 BI | 0.03*** |
| H8: C 🡪 BI | -0.03 |
| H9: E 🡪 BI | 0.02* |
| H10: A 🡪 BI | 0.02* |
| H11: N 🡪 BI | 0.05*** |

*Table A2.* Between-country analysis predicting behavioral intention to use CondAVs, direct effects

| **Hypothetical path** | **U.S.** | **UK** | **FR** | **HU** | **DE** | **CN** | **BR** | **JP** | **RU** |
| --- | --- | --- | --- | --- | --- | --- | --- | --- | --- |
| ***R^2^* in BI** | **0.91** | **0.87** | **0.89** | **0.90** | **0.83** | **0.86** | **0.87** | **0.78** | **0.83** |
| H1: PE 🡪 BI | 0.25  ** | 0.28  *** | 0.17  * | 0.14  * | 0.09 | 0.73  *** | 0.43  *** | 0.26  *** | 0.46  *** |
| H2: FC 🡪 BI | 0.03 | 0.07  ** | 0.05  * | 0.06  * | 0.04 | -0.01 | -0.02 | 0.01 | 0.06 |
| H3: SI 🡪 BI | 0.53  *** | 0.33  *** | 0.37  *** | 0.29  *** | 0.32  *** | -0.08 | 0.33  *** | 0.33  *** | 0.16  * |
| H4: HM 🡪 BI | 0.06 | 0.05 | 0.06 | 0.06 | 0.16  *** | -0.05 | 0.00 | 0.03 | 0.05 |
| H5: TRU 🡪 BI | 0.16  *** | 0.33  *** | 0.33  *** | 0.54  *** | 0.44  *** | 0.27  *** | 0.28  *** | 0.39  *** | 0.29  *** |
| H6: DE 🡪 BI | -0.00 | -0.04 | -0.09  ** | -0.09  * | -0.03 | -0.32  * | -0.01 | 0.03 | -0.03 |
| H7: O 🡪 BI | 0.03 | 0.04  * | 0.10  *** | 0.01 | -0.02 | 0.06 | 0.05* | 0.02 | -0.00 |
| H8: C 🡪 BI | 0.03 | 0.03 | -0.03 | 0.03 | 0.01 | -0.03 | 0.01 | 0.10  *** | -0.06  * |
| H9: E 🡪 BI | 0.05  * | 0.03 | 0.03 | 0.01 | 0.02 | 0.11  ** | 0.03 | 0.03 | 0.04 |
| H10: A 🡪 BI | -0.01 | -0.03 | 0.01 | 0.03 | 0.05  * | 0.05 | 0.01 | -0.02 | 0.04 |
| H11: N 🡪 BI | 0.05  * | 0.02 | 0.04  * | 0.06  ** | 0.05  * | 0.13  ** | 0.02 | -0.03 | -0.03 |

*Note: R*^2^ denotes the variance accounted for in the predicted variable. Presented next to *R*^2^ are the standardized beta coefficients β, and the significane levels **p* < 0.05; ***p* < 0.01; ****p* < 0.001. No significance level indicates a non-significant relationship.
